# Peer researchers in NHS research: approved in principle, undermined in practice?

**DOI:** 10.1186/s40900-025-00708-0

**Published:** 2025-06-17

**Authors:** Bryher Bowness, Peter Bates, Abnash Chauhan, Yasma Osman, Tanya Shlovogt, Vanessa Lawrence

**Affiliations:** 1 Institute of Psychiatry, Psychology and Neuroscience, King’s College London, London, UK; 2 Peter Bates Associates, Nottingham, UK; 3 Joint Research & Development Office of South London and Maudsley NHS Trust and Institute of Psychiatry, David Goldberg Centre, London, UK; 4 Health Service & Population Research Department, David Goldberg Centre, De Crespigny Park, Denmark Hill, London, SE5 8AF UK; 5 Independent Researcher, Manchester, UK

**Keywords:** Marginalisation, Coproduction, Patient and public involvement (PPI), NHS, Health and social care research, Peer researchers, Ethics, Inequality and discrimination

## Abstract

**Background:**

Despite the increasing support and expectation for involving people with lived experience in healthcare research in England, challenges persist when navigating organisational structures. This can result in unintentional exclusion and disempowerment.

**Main body:**

This reflective case report describes the experiences of a doctoral student working with their Research & Development department to determine the checks required for a peer researcher (without an employment contract) to co-facilitate focus groups with National Health Service (NHS) users. Despite best efforts, the absence of clear guidance about the necessary processes for obtaining her approval documents (known as ‘Research Passports in the UK) resulted in delays, distress, and the peer researcher resigning. Current procedural complexities of facilitating peer research in the NHS may be perpetuating rather than addressing systemic inequality. Reflecting together as academics and research governance departments, we hope to illuminate steps that can be taken in advance to mitigate future harms.

**Conclusions:**

By taking shared responsibility for what needs to be changed, we hope to open a dialogue that will create collaborative and consistent practices aligned with the principles and aspirations of involving peer researchers.

## Abstract (plain language summary)

There is growing support for involving the public in healthcare research, because ‘peers’, i.e., people with lived experience of the topic, bring important insights. However, when people are not employed by a university or the National Health Service (NHS), the processes they may have to go through to help conduct the research may be very complicated. This can discourage them from becoming involved. This paper describes the attempts of a research student (BB) and a family carer peer researcher (YO) to design and conduct a study collaboratively. They planned to hold online discussion groups with family carers. To do this, both the peer researcher and the research student had to first apply to the local NHS Research & Development department for a letter demonstrating that they had undertaken the relevant checks to meet with NHS service users. However, this department was unsure which checks were needed for the peer researcher. National guidelines and advice from others working in the field were unclear or contradictory. Everyone worked together for 6 months to find this out, but eventually, YO resigned. Our story reveals how systems can make it difficult for members of the public to join a research team, particularly those who are not part of established organisations. We aim to raise awareness of what must be changed to reduce harms. All those working in research have a shared responsibility to work with each other and with the public to design and promote clearer processes, to make research more equal and inclusive in the future.

## Introduction

“Research engagement that seeks to disrupt power dynamics and effect social change can be demanding and complex, so it is not surprising that mess can sometimes ensue” [1].

Participatory research is not a new approach, it has a long history and is common practice in some fields. There are many different ways that those outside academia with lived experience of the issue can be involved. In health research, recommendations to conduct research ‘with’ rather than ‘for’ the public [2] have led to increasing public involvement, albeit most often in advisory capacities [3]. Setting out on my PhD, I aspired to coproduce my research; to take an “approach in which researchers, practitioners and the public work together, sharing power and responsibility, from the start to the end of the project” [4]. We hoped to do this by involving those who have lived/are living through the matter being investigated as ‘peer researchers’ [5]. Peer researchers bring new insight and a wealth of expertise-by-experience to the research process and outcomes. They may be involved in any, and ideally all, of the research stages, e.g. as co-applicants, partners in data collection, analysis, or coauthors. In our specific case, we describe and reflect on the unanticipated complications in obtaining the required permissions for our peer researcher to join our researcher in cofacilitating recorded online focus groups.

Our participants were staff and attendees of courses promoting mental health and recovery provided by Recovery Colleges that, in many cases across England, are funded by the National Health Service (NHS). Some of the challenges we encountered may be particular to procedural issues NHS settings, as they echo the bureaucratic barriers faced by peer researchers in other studies [6, 7] dating back to 2007 [8]. This case study is also situated in a history of institutionalized racism in England, which academic and healthcare organizations are only now beginning to strategically address (e.g. Patient and Carer Race Equality Framework [9]**)**. But we believe there may be some resonance with experiences globally for how systems governing peer research can inadvertently perpetuate power relations and exclusion.

There is often a reluctance to write about failure in participatory research. Perhaps there is a fear of discrediting rather than encouraging the approach, undermining the credibility of the research outputs [10], appearing to point the finger, or unintentional censorship arising from publishing pressures and perceived risks of litigation 1. However, to create change, it is important to reflect on the challenges and potential harms of involving the public within the current institutional context [11]. For participatory approaches to be embedded into the systems that healthcare research relies upon, it is essential that we disseminate the learning resulting from efforts to drive this forward. Our aim is not to assign blame but to identify practices that need to change and, most importantly, to open-up dialogue to create collaborative solutions.

## Academic’s story

As a result of the negative experiences that we will describe, the peer researcher left her role and all future research projects. She declined the offer to pursue an official complaint or to write her own account but instead urged the doctoral student (BB) and colleagues to share the story. The following paragraphs were therefore drafted by the researcher and then endorsed by the peer researcher as an accurate representation of events and the issues she perceived.

“For my PhD, I set out to explore experiences of family, friends, and supporters (hereon known as family carers) who had attended Recovery Colleges, establishments offering a coproduced, educational approach to promoting mental health and empowerment [12]. Inspired by this ethos, I set out to design and conduct my project with a family carer outside of academia. I discussed my plans with the Lived Experience Advisory Panel (chaired by coauthor PB) for a larger existing research programme in my department, RECOLLECT 2 [13]. One member of this panel (YO) expressed a strong interest in my research, and after completing a brief application form and terms of reference for the role, she was subsequently appointed as a peer researcher on the study. The role was voluntary, recognised through gratitude payments rather than a contract of employment. My systematic review [14] found that inviting family carers to design their role ensures that it is feasible and rewarding for them and helps challenge traditional power imbalances. The peer researcher was especially motivated to codesign the study and cofacilitate focus groups, interested to hear peoples’ firsthand accounts, but also to use her experiences to relate to participants. Family carer facilitators were reported to help participants feel more comfortable and bring new awareness to conversations [15, 16]. Given that I had read examples of peer researchers conducting unchaperoned face-to-face interviews with participants who might be deemed ‘vulnerable’ (e.g. [17]), I assumed that YO’s comparatively lower risk role of facilitating groups online would be possible.

**Fig. 1:**
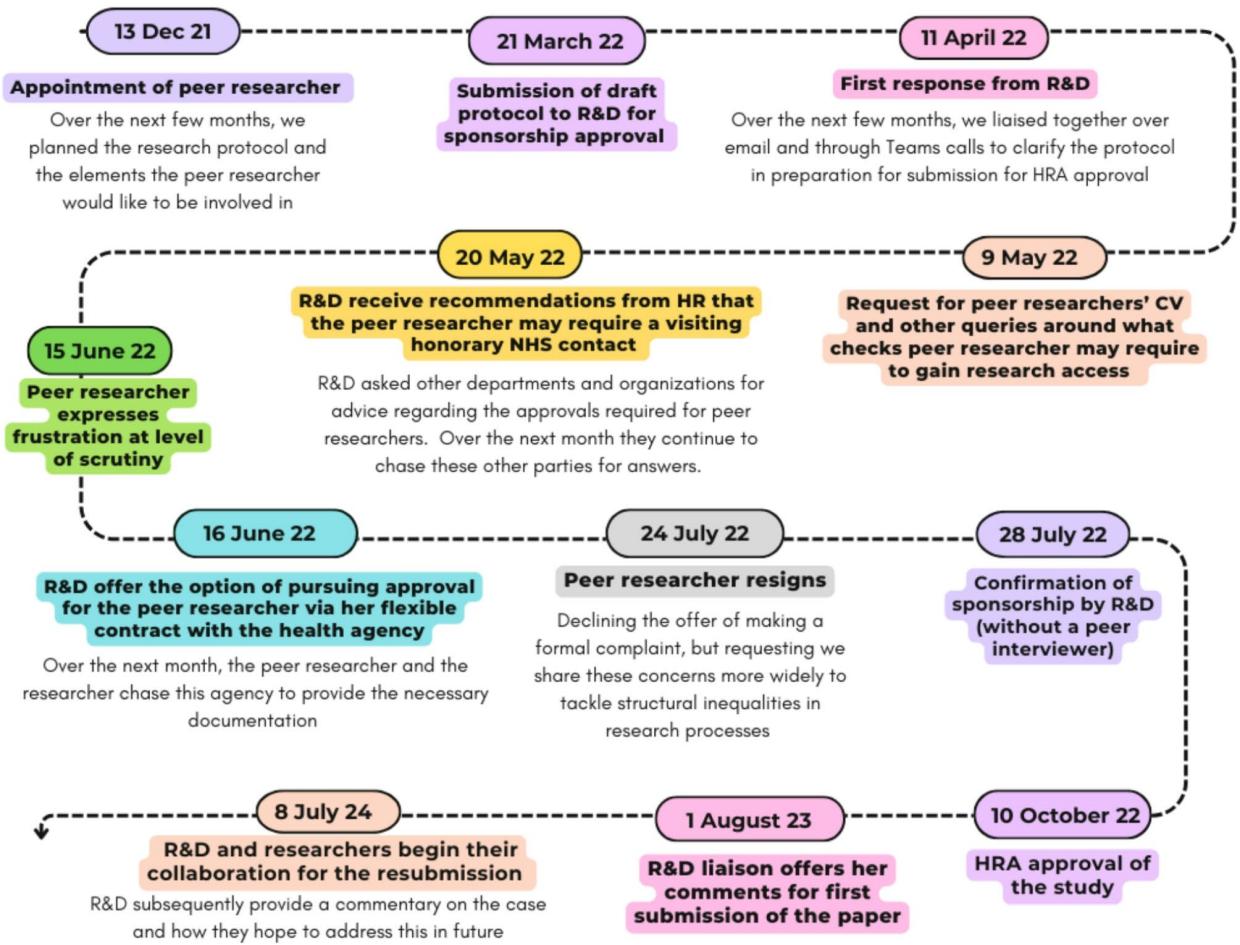
Timeline of inquiry for the required permissions for the peer researcher’s access

As my research was to be conducted at sites partly funded by the NHS, I was required to first submit our protocol to the local Research and Development (R&D) Department. Each NHS Trust has an R&D Department that oversees the research governance process for any study hoping to recruit participants using their services and provides researchers with sponsorship^2^ required to apply to Research Ethics Committees (RECs). Our small R&D team was very responsive and conscientious. They instantly assigned me a liaison who gave a thorough response to my submission within weeks and ensured ongoing dialogue and transparency throughout. [see Fig. 1]. My liaison kept me updated with her progress, offering frequent calls to talk through issues where she was unclear, and she sought potential options to overcome the barriers we faced together.

Academic researchers must usually be issued a Research Passport and Letter of Access, a mechanism that provides evidence of the pre-engagement checks undertaken on the researcher in line with the NHS Employment Check Standards [18]. Some projects also require researchers to undergo a DBS (Disclosure and Barring Service checks of the history of criminal convictions). Our R&D liaison explained that their department had not previously reviewed studies where peer researchers were involved in the data collection, so they did not know what processes of approval YO would need to go through to be approved for access to NHS service users.

Our R&D liaison sought advice from the University Human Resources team, the NHS Trust Transactional Services Advisor, and the national body, which oversees ethical approval (Health Research Authority, HRA). These parties often took time to respond, and provided responses that contradicted suggestions from other organisations. My supervisors also met with the R&D manager, trying together to find a feasible way to involve the peer researcher in the data collection. To aid the R&D team, I sought other examples, e.g., [8, 19], and spoke to other academic researchers who were experienced in participatory research in NHS settings.

In some instances, R&D departments agree to ‘sponsor’ a study, which subsequently allows researchers to apply to HRA for overall ethical approval before the checks on individual researchers have been granted. For example, our peer researcher was also on an advisory panel for the RECOLLECT 2 project, which planned in a later workpackage to involve our peer researcher in conducting interviews and focus groups with users of recovery colleges across multiple NHS sites in England. This workpackage for RECOLLECT 2 had already received a favourable opinion from an NHS REC approving their study protocol. However, this application was sponsored by a different NHS Trust, and they had not yet gained local access to NHS research sites. RECOLLECT 2 has since found that access requirements for peer researchers are variable across different Trusts; some did not require any approval systems if a research employed research assistant was present, whereas at other sites checks are still underway years later. In our situation, our R&D liaison felt that because she was not receiving any direct answers relating to what YO would need to be allowed involvement in data collection, the level of uncertainty was such that we should wait before submission for HRA approval. In our instance, delays occurred before sponsorship, but others described similar experiences when applying for research passports to initiate studies at a site, e.g., [7]. We could not provide the peer researcher with a clear timeline, adding to confusion and a lack of control.

Initially, the R&D Department asked for the peer researcher’s curriculum vitae (CV), with justification that this was needed for all members of the research team. Unaware of any alternative option, I interpreted this as the requirement of a traditional CV. While this may be intimidating to some peer researchers, our peer researcher was proud of her skills and achievements and happy to share them. However, she was subsequently asked for further evidence and checks before she was able to cofacilitate the focus groups. She told me that she felt disrespected and discriminated against as a Black African Muslim woman who did not have ‘letters after her name’ (which the traditional CV values). While we discovered later that this R&D Department asked for the CV as evidence of a current substantive employer, it naturally conveyed to our peer researcher a questioning and subsequent dismissal of her competencies to conduct research.

One suggestion from my peer researcher was that the healthcare agency she had a flexible contract with may be able to provide the necessary references for a Letter of Access, which our R&D Department said may be adequate in this instance. Unfortunately, after over one month of myself and the peer researcher calling and emailing various departments in the agency, we still had no response from the agency to this request. Another option proposed by the R&D team was that our peer researcher would need to apply for an honorary contract with the NHS. We were warned that the process of obtaining this could take many months, requiring numerous references, identity documentation checks, and occupational health clearance. Having already responded to numerous requests for information, the peer researcher did not have time to undergo the further administrative burden. Moreover, neither the peer researcher’s employment status nor the research activities she was hoping to undertake did not fall into the definition of activities regulated by honorary contracts [18, 20]. Of course, some level of accountability needs to be in place for all researchers. However, given the focus groups were online, and I would accompany the peer researcher throughout, the demands required for her to obtain an honorary contract seemed to be greater than any mitigation of risk they might provide.

The arguably disproportionate requirements placed on the peer researcher, along with the long period of uncertainty and repeated requests for more personal information over several months, understandably left her feeling heavily scrutinised. She identified the process as a systemic barrier to her inclusion in the project. My naivety to the processes meant that I could not provide the peer researcher with sufficient alternative explanations. After four months of back-and-forth efforts to determine the peer researcher’s route to apply for a Research Passport, and an enormous amount of work on behalf of the R&D team, we all felt frustrated. Understandably, the peer researcher eventually resigned. She had already given up large amounts of time, an especially precious commodity for a family carer, and said she wanted to prioritise activities that contributed to meaningful change. YO believed that the opportunity would allow her voice to be heard in research but had found herself responding to emails and going through unexpectedly long bureaucratic processes^3^. The burdens and barriers faced by the peer researcher communicated to her that she was not an equal member of the team. She concluded, “I’m either all in, or all out” and resigned from her roles on both research projects with our department.

YO was now deterred from all involvement, thus the enthusiasm and expertise that she would have brought to future research has also been lost. Mostly, I felt responsible, as I had brought the peer researcher into the experience, and I had not warned her about the potential difficulties, which led to her subsequent distress. The progress of the PhD was significantly delayed, and I was disappointed that the study was no longer fully coproduced. I remained dedicated to participatory research, and became involved in groups working towards developing new systems that might reduce some of the bureaucratic barriers. However, with time of the essence, I felt it would be easier to conduct my next study outside of the NHS.”

## Discussion

It was the peer researcher’s integrity and bravery in setting out her boundaries that exposed the inequalities and flaws in the current system, on which we will now reflect.

Despite encouragement in best practice guidance [21], it seems that involving the public *throughout* the process is less common in health research than we believed. There remains “a significant gap between what policy espouses and what transpires in real-world practice,” p799 [3]. Indeed, fewer than 20% of papers in mainstream medical journals demonstrated substantial public involvement [22]. Tokenistic practices [23], where peer researchers have a predefined role and limited input into decision-making, can replicate and act to mask oppressive systems in society [24]. It was astute and admirable that our peer researcher refused to be involved in ways that she felt reinforced existing hierarchies. The ownership she initially felt over the project through codesign was eroded, as it became clear that her involvement was frustrated by the bureaucratic barriers of institutional structures, some of which we will now discuss.

Firstly, compared with participatory research outside the NHS, additional safeguards may be considered necessary where participants are patients of healthcare services. ‘Research in the NHS – HR Good Practice Resource Pack’ states that research within the NHS undertaken by non-NHS staff “raises issues about responsibility, accountability, patient safety and duty of care” p2 [18]; thus, arrangements and clarity around responsibilities and liabilities are needed. R&D Departments play a role in the duty of care, holding responsibility for minimising the likelihood of research leading to harm to NHS patients. Peer researchers are hard to classify because they have lived experience like patients do, but their roles and responsibilities are more similar to those of staff without the same contractual obligations to an accountable organisation. This liminal space, where different perspectives may be held on the relative importance of these overlapping identities, is a breeding ground for confusion.

To grant site access, local R&D departments act independently, predominantly using discretion and local policies to authorise research. Guidance for the responsibilities of research sites and sponsors, such as the HRA UK Policy Framework [25], can be vague in terms of the required authorisation mechanisms to obtain permission to conduct research with patients, and processes are not standardised. It is unclear whether these findings would apply to peer researchers who are salaried. Navigating this complexity results in diverse arrangements for peer researchers in NHS settings across England: Research Passports and Letters of Access, Honorary Employment Contracts, registration on volunteer databases, Service Level Agreements, or no formal documentation at all [20]^4^. While we recognise the benefits of context-specific knowledge and the flexibility to exercise judgement, many governance departments are unfamiliar with how to regulate participatory practices safely, ethically and proportionately [6]. Naturally, they may tend towards more cautious and thorough practices, which can be incidentally more costly or exclusionary. This poses a further risk that researchers will circumvent these safeguarding processes entirely to involve peers.

When peers are contracted or are members of official organisations, the processes for them to gain access to participants are more clearly defined in national guidance, perhaps because R&D departments are able to delegate liability for any potential misconduct. However, other motivators for ethical conduct and accountability exist beyond legal and institutional realms (such as moral and relational assurances), which should be explored.

The same checks are often much easier for a university researcher than for a peer researcher, who is unfamiliar with the systems and may face additional barriers to obtaining required documents. Inequalities inbuilt into accessing certain activities accentuate the power disparities within the research team, and some peer researchers described the experience of applying for authorisation in NHS research as a ‘rite of passage’ to symbolise acceptance into academia [7].

YO’s lived experience as a Black Muslim woman provided an invaluable counterpoint to the academic researcher’s privilege and bias, which would have brought insight and sensitivity to facilitating the focus groups. Systems favouring those with contractual employment, societal connections, not to mention time and literacy to fill out long forms [11], limits involvement to those who have been who have been socialised into existing research norms [26], rather than hearing voices of those ‘beyond the usual suspects’ [27]. The requirements of the honorary contract application (an option suggested in our case), exclude anyone who is denied the right to work in the UK, and DBS checks are more difficult for those without official documents (such as driving licences and passports). Equitable systems do not treat people the same, but rather allow everyone the same opportunities, if needed, through reasonable adjustments. Current practices disproportionately disadvantage peer researchers from marginalized communities, contributing to the lack of racial diversity within public contributors [28, 29].

The bravery of the peer researcher to speak out and set boundaries sensitised the whole project to the entrenched inequality and multiple processes by which people may be silenced. Passionate about raising awareness of the issues of discrimination faced by minorities when they encounter health services, the peer researcher questioned whether the administrative hurdles she faced were indicative and perpetuating of structural racialized inequalities across academic institutions and health services [30]. The responsibility to address these issues should not, however, continue to lie with those who have faced discrimination. Like the PhD student in our example, academics are often naïve to the lasting impact of community stakeholders’ prior experiences of these inequities [31]. But these inevitably shape how peer researchers perceive current involvement [32]. Those from marginalised communities often experience mistreatment by authorities, so they may interpret requests for documentation as indicative of suspicion [33]. Negative experiences during the early stages of research partnerships, where a significant amount of time should be put into building trust [31], will likely impact their perceptions of researchers and academia as a whole [34]. This was evident in our narrative, where YO subsequently pulled out of her involvement in her other projects too as a result of her experience in the onboarding stages.

We suggest that bureaucratic safeguards applied to our peer researcher, intended to reduce risks to participants, could unintentionally communicate a mistrust to peer researchers from racialized backgrounds who have frequently experienced suspicion and discrimination by authorities in wider society^5^. Whilst our story is not one of motivated racial exclusion, the distress experienced in circumstances where this might be perceived will have the same consequences. To avoid retraumatising survivors, it is essential that the conditions of involvement do the opposite of the initial abuse [36], which in this case includes long history of institutional racism and colonialism. We are inviting peer researchers into our environment, which they may find unfamiliar and intimidating, so ensuring that this space is safe and empowering is our responsibility [11]. Rather than denouncing the efforts of colleagues, advocates of coproduction should collaborate to provide sufficient governance processes for peer researchers.

## R&D colleagues’ commentary

**“**The Joint R&D Office of South London and Maudsley (SLaM) NHS Foundation Trust and Institute of Psychiatry, Psychology & Neuroscience (IoPPN) reviewed this paper, which describes the experience of a research team in 2022 trying to navigate the complexities around permissions for public/peer researchers’ involvement in research. We then held discussions with the researchers about the events that took place and explored the issues this raised, before writing this commentary. The particular staff members involved in 2022 had moved on. However, because the case was shared at the time in our weekly department facilitation meetings (held routinely to enable continuous collaborative reflection to inform our practices), we were able to continue the conversation when approached by the researchers a few years later.

The aim of this current collaboration has been to work together to reflect on this experience and the wider systematic factors contributing to it, as well as to consider implications and process improvements. Following reviewing the case and involvement with this paper, we have reconsidered the need to request CVs from all members of the research team, as we appreciate that this may be a key deterrent for members of the public in becoming involved. We are also in the process of adapting our Standard Operating Procedures for Research Passports as a result of these reflections and insights.

The work we are doing resulting from this example to reduce barriers to peer research is part of a wider commitment we have made as a department and NHS Trust, working towards inclusive and antiracist practices in research. This underlying strategic drive to address inequalities in mental healthcare enables the recognition of how systems perpetuate structural discrimination in everyday processes. SLaM and IoPPN have an existing strategy to increase diverse representation among their academic workforce. They also host a Building Race Equity and Diversity (BREaD) in research network. Established in 2022, this network facilitates partnership working, bringing in numerous stakeholders from across South London healthcare services and the community [37]. BREaD has carried out projects to understand how we can increase inclusivity in research participation. For example, we have made it mandatory for all studies to collect demographic information to enable ongoing monitoring for who may be underrepresented amongst our participants. Considering the case, our R&D department appreciates that it is imperative to improve processes wherever possible to encourage diverse groups to partake in research, not only among those recruited to participate but also among peers and academics who make up the research team.

Learning from the example in this report would have been limited without the collaborative reflection and ongoing dialogue between our department and the academic researchers involved. Recognising this, we have secured a Research Impact and Culture Award, which enables regular drop-in joint lunches between professional services and academics. One of the aims is to sustain a space for this dialogue, reduce misunderstandings and creating a shared culture of learning.

## Implications for future work

Only through reflecting on these issues collaboratively with members of the R&D have we become aware of relevant guidance and its rationale, and potential solutions. In this section, the academic authors suggest some small steps towards creating fairer access for peer researchers (see Table 1).

There are many declarations of commitment to coproduction but a comparative absence of guidance for its practice [38]. To create clearer processes for peer researchers, national bodies, such as the HRA or the NIHR, should publish specific and contemporary recommendations for how to involve peer researchers in data collection with service users. Coauthor PB has developed a useful guide to this [20]. Whilst also minimising ambiguity, arrangements must also be flexible enough to encompass the diversity within participatory practices. This could be achieved by collating and sharing case-studies within departments, as described in our R&D Commentary, and disseminating these more widely, for example development of practical toolkits for Community-Based Participatory Research [39]. Clear procedures encourage academic researchers to embark on coproduction, enable communication for what peer researchers might expect from the outset, and promote more positive risk-taking among local R&D departments.

We acknowledge that the competency of anyone undertaking research with people should be examined but advocate for new, more inclusive and proportionate ways of achieving this for peer researchers from outside of academia [20]. Designing processes with communities marginalised by traditional systems will enable them to communicate competencies previously overshadowed by academic bias. We need to raise awareness that alternative CV templates can be offered (e.g., ‘My Involvement Profile’ [40]). Both peer researchers and academic researchers should have an opportunity to share with R&D departments the specific skills and competencies needed to complete the peer researcher role safely so that documentation and other checks are directly relevant to the individual role rather than arbitrary. Coauthor PB proposed an example framework to understand the different roles undertaken by public contributors [41].

NIHR guidance [42] advises that risk assessments should be carried out to ensure the safety of peer interviewers. We would argue that researchers should consider the potential distress that might be experienced during the initial administrative phases, and planning risk mitigation strategies such as providing wider team training about public involvement. We must recognise that governance systems may lead to unintended harms. Future inquiry into the reasons peers end their involvement may reveal systems responsible for harm or inadvertent discrimination that can be changed. Creating safe spaces for peer and academic researchers and R&D departments to share and build on experiences of failure, underpinned by the kind of respect and transparency that we received during our partnership with our R&D department would enable this transformation.

Cook’s methodological framework of reflecting on the ‘mess’ involved in participatory research unearths its complex ethical issues and unintended effects [43, 44]. Although the minutiae of legislation and guidelines rarely appeal to academics, we must engage with it to prevent bureaucracy inhibiting our aspirations to conduct ethical and emancipatory research. Brushing over conflicts to achieve a quick win will perpetuate difficulties in the long run [26]. Approaching R&D colleagues prior to starting an NHS study to clarify how peer researchers may be included will reduce the risk of involving someone in a harmful process or curtailing discussions to address imminent research deadlines. The inclusion of peer researchers in these debates will help professionals gain awareness of the ongoing influences of intersectional structural inequalities.

## Conclusion

The narrative described above illustrates some of the difficulties resulting from the lack of consensus and clarity regarding processes for peer researchers. Researchers are unable to inform peers about what to expect, or the rationales behind demands, which can be disempowering or exclusionary. A lack of familiarity with participatory research methods could lead departments to default to irrelevant, disproportionate or biased criteria for approval. This can reproduce the wider societal inequalities participatory research aspires to address. Researchers and peers will either be deterred from engaging in participatory research in the NHS, or will look for ways to bypass important protective measures, unless we create facilitative governance mechanisms for including the public. We need to collaboratively reflect on these failures openly and urgently begin shared discussions to create guidance that aligns practice with principle.

**Table 1:** Summary of recommendations

Recommendations	
Researchers	• Proactively engage with your R&D department to clarify local processes for granting Research Passports for peer researchers (if possible, before seeking funding, sponsorship and access). • When you encounter successes, difficulties or failures during your participatory endeavours, publicise these.
Health Research Authority (and other national regulatory bodies)	• Collate disparate advice and best practice examples/practical guidelines of processes and distribute these to local governance teams.
Research & Development Departments	• In advance of requests, provide a defensible mechanism by which suitable peer researchers can apply for access to NHS patients. Make it proportionate and relevant to the role and level of risk, whilst being accessible and making any reasonable adjustments for those who may face additional barriers in bureaucracy.
All	• Take responsibility for and engage in collaborative scrutiny of systems that may be causing harm in the research world • Take considered and engaged approaches to the development of alternative, trauma-informed and inclusive practices.

## Data Availability

No datasets were generated or analysed during the current study.

## Footnotes

1 something we experienced during previous submissions of this article.

2 An organisation agrees to take overall responsibility to ensure that proportionate and effective arrangements are in place for the research project to take place.

3 For those conducting research as part of their employed role within an organisation, extensive administrative demands are more expected almost as inevitabilities.

4 These are some examples of different practices, which were collected through literature searches and discussions with colleagues asking for further details of the governance processes required in their projects.

5 This may be particular to our UK context, where black people have historically received discriminatory treatment by authorities, such as the police stop and search policies, which has been repeatedly covered up by institutions ([35]. Bowling B, Phillips C. Disproportionate and discriminatory: Reviewing the evidence on police stop and search. The modern law review. 2007;70(6):936 − 61).

## Abbreviations

R&D Department: Research & Development Department
NHS: National Health Service
PhD: Doctorate of Philosophy
REC: Research Ethics Committee
HRA: Health Research Authority
RECOLLECT 2: Recovery Colleges Characterisation and Testing 2 (research project name)
CV: Curriculum Vitae
NIHR: National Institute for Health and Care Research
IoPPN: Institute of Psychiatry, Psychology and Neuroscience
SLaM: South London and Maudsley
DBS: Disclosure and Barring Service
